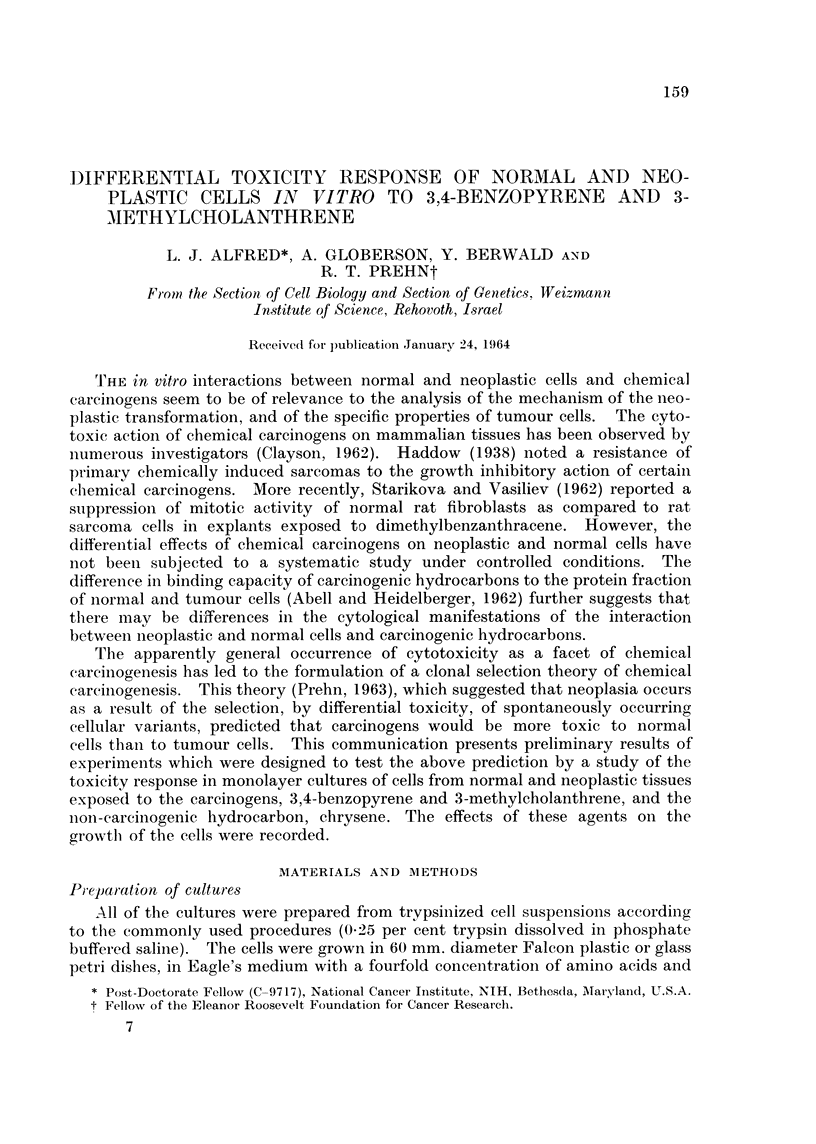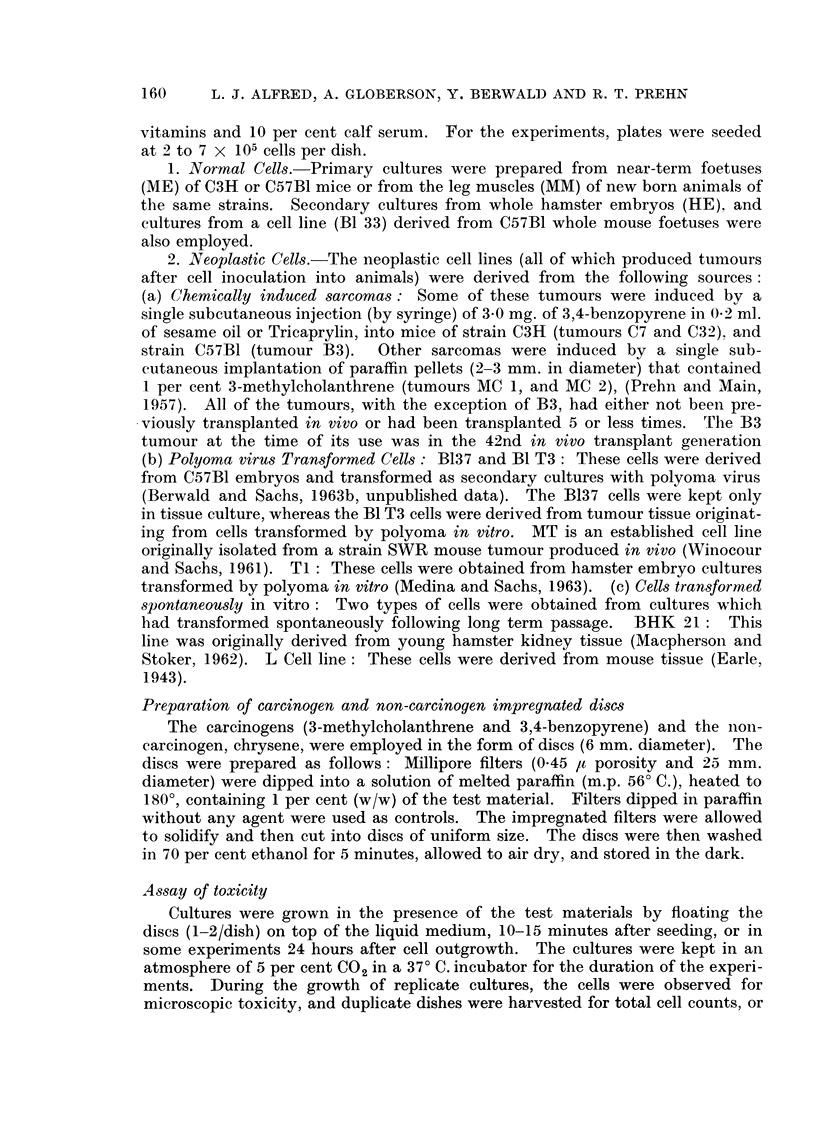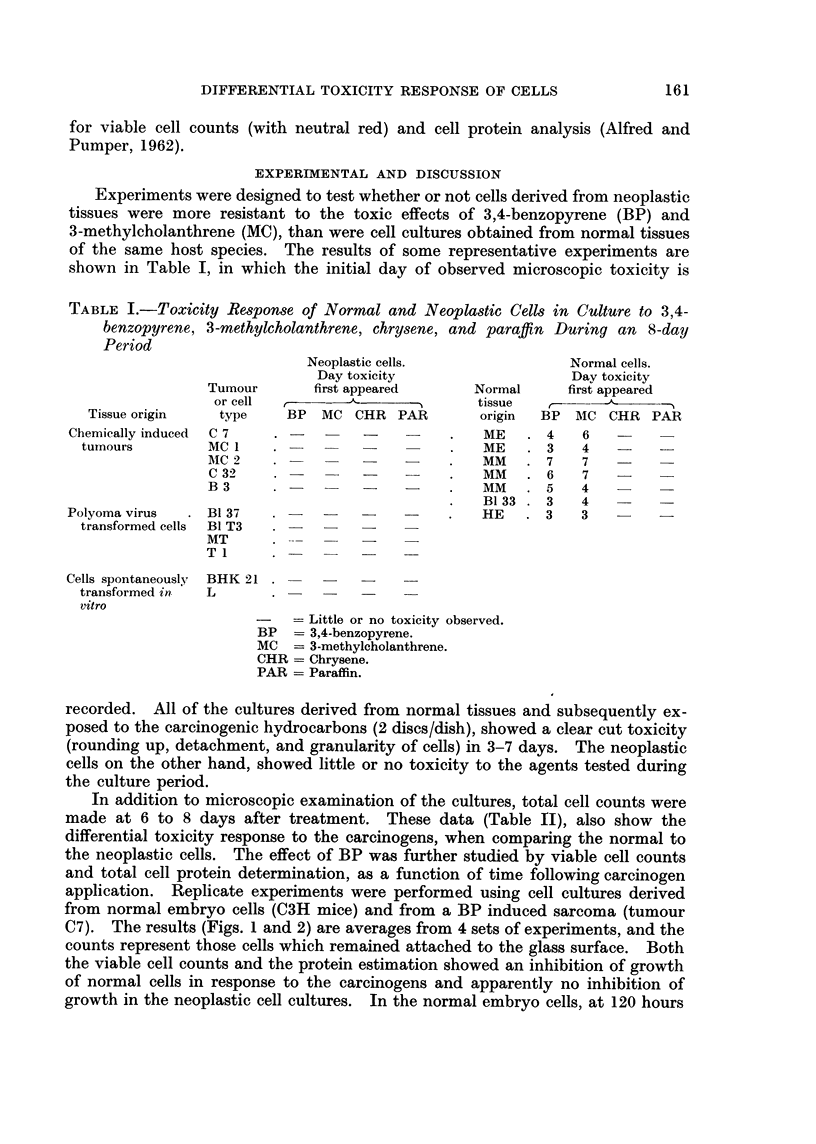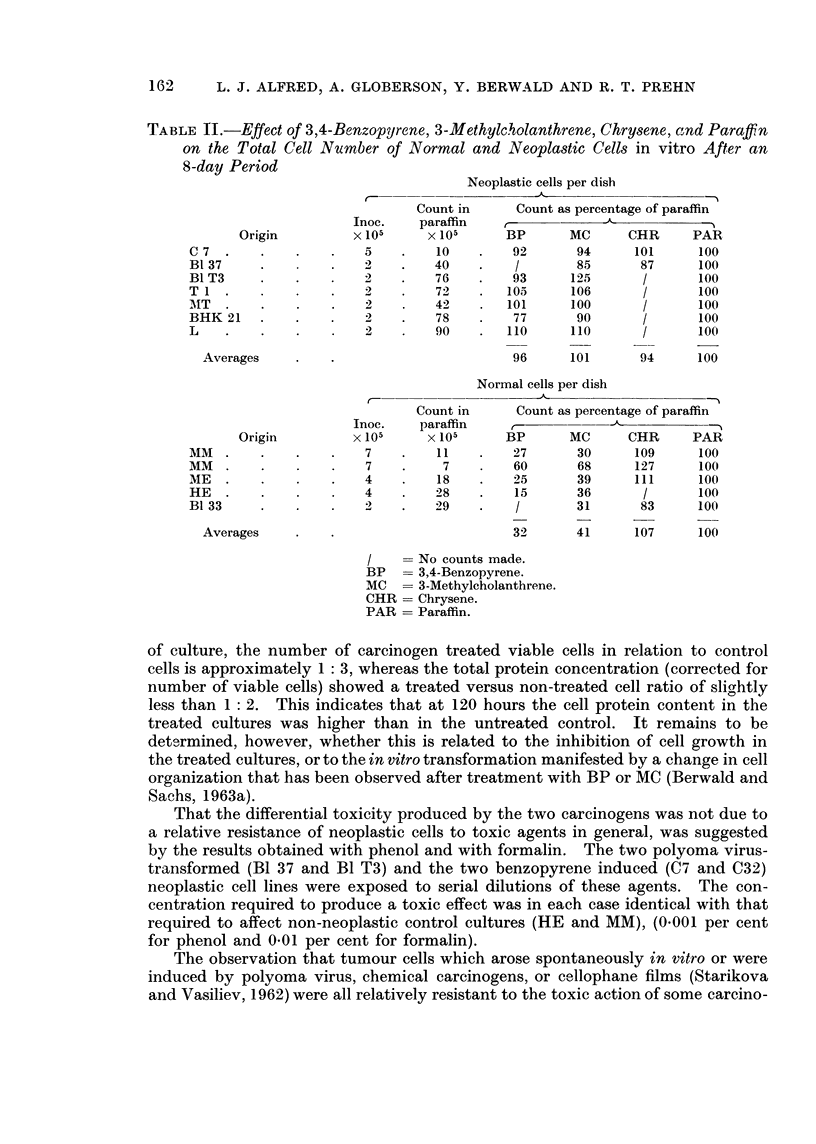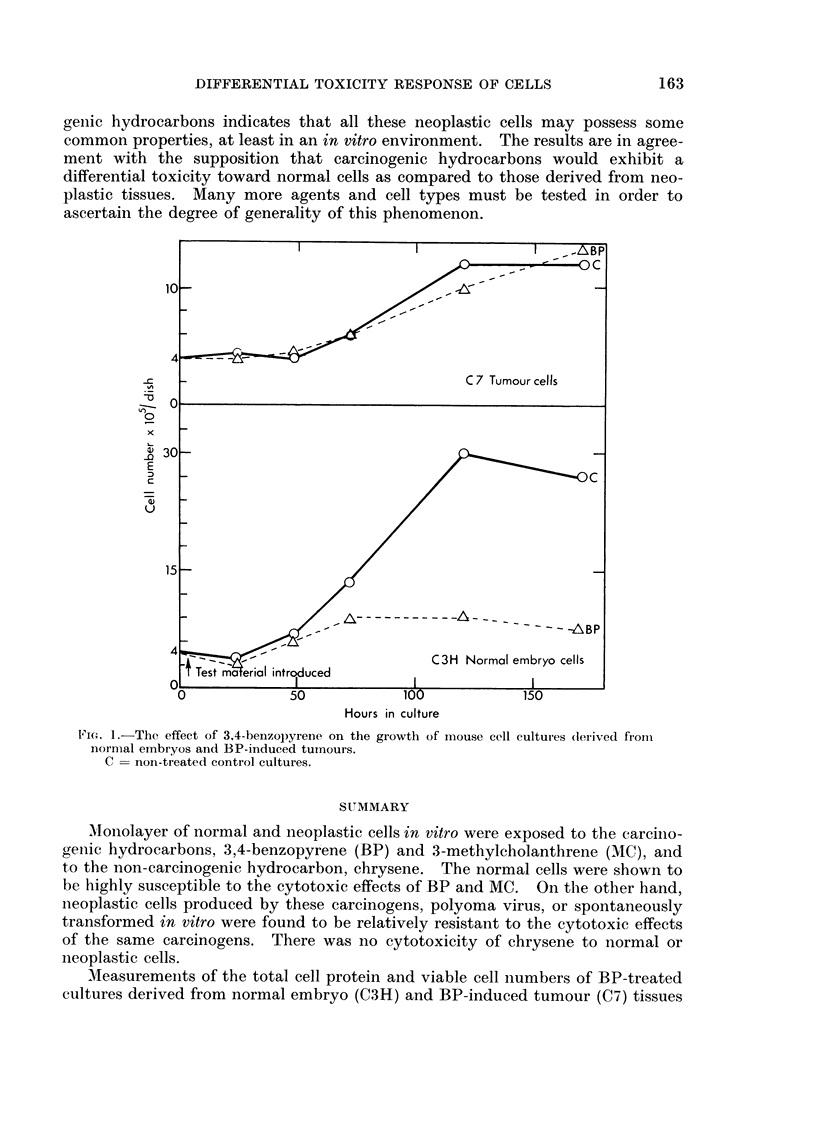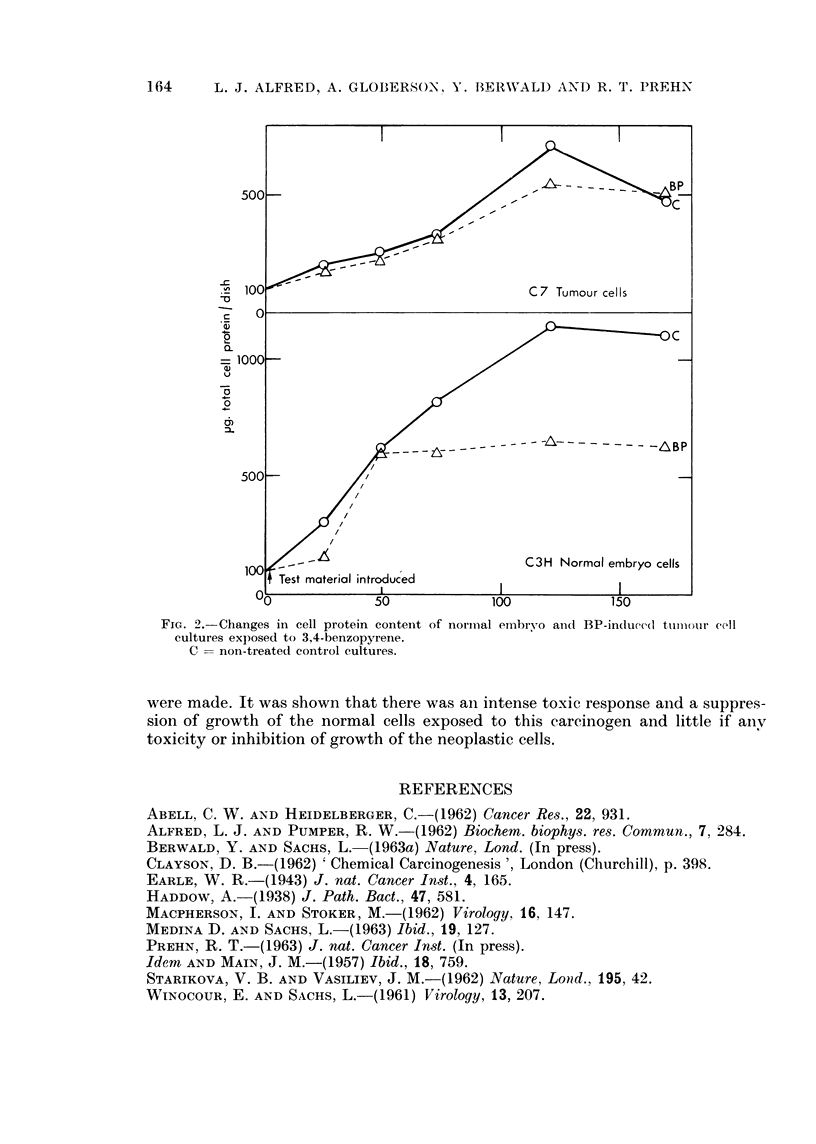# Differential Toxicity Response of Normal and Neoplastic Cells In Vitro to 3,4-Benzopyrene and 3-Methylcholanthrene

**DOI:** 10.1038/bjc.1964.18

**Published:** 1964-03

**Authors:** L. J. Alfred, A. Globerson, Y. Berwald, R. T. Prehn


### DIFFERENTIAL TOXICITY RESPONSE OF NORMAL AND NEOPLASTIC CELLS *IN VITRO* TO 3,4-BENZOPYRENE AND 3-METHYLCHOLANTHRENE

L. J. ALFRED*, A. GLOBERSON, Y. BERWALD AND R. T. PREHN†

*From the Section of Cell Biology and Section of Genetics, Weizmann Institute of Science, Rehovoth, Israel*

Received for publication January 24, 1964

THE *in vitro* interactions between normal and neoplastic cells and chemical carcinogens seem to be of relevance to the analysis of the mechanism of the neoplastic transformation, and of the specific properties of tumour cells. The cytotoxic action of chemical carcinogens on mammalian tissues has been observed by numerous investigators (Clayson, 1962). Haddow (1938) noted a resistance of primary chemically induced sarcomas to the growth inhibitory action of certain chemical carcinogens. More recently, Starikova and Vasiliev (1962) reported a suppression of mitotic activity of normal rat fibroblasts as compared to rat sarcoma cells in explants exposed to dimethylbenzanthracene. However, the differential effects of chemical carcinogens on neoplastic and normal cells have not been subjected to a systematic study under controlled conditions. The difference in binding capacity of carcinogenic hydrocarbons to the protein fraction of normal and tumour cells (Abell and Heidelberger, 1962) further suggests that there may be differences in the cytological manifestations of the interaction between neoplastic and normal cells and carcinogenic hydrocarbons.

The apparently general occurrence of cytotoxicity as a facet of chemical carcinogenesis has led to the formulation of a clonal selection theory of chemical carcinogenesis. This theory (Prehn, 1963), which suggested that neoplasia occurs as a result of the selection, by differential toxicity, of spontaneously occurring cellular variants, predicted that carcinogens would be more toxic to normal cells than to tumour cells. This communication presents preliminary results of experiments which were designed to test the above prediction by a study of the toxicity response in monolayer cultures of cells from normal and neoplastic tissues exposed to the carcinogens, 3,4-benzopyrene and 3-methylcholanthrene, and the non-carcinogenic hydrocarbon, chrysene. The effects of these agents on the growth of the cells were recorded.

### MATERIALS AND METHODS

### *Preparation of cultures*

All of the cultures were prepared from trypsinized cell suspensions according to the commonly used procedures (0·25 per cent trypsin dissolved in phosphate buffered saline). The cells were grown in 60 mm. diameter Falcon plastic or glass petri dishes, in Eagle's medium with a fourfold concentration of amino acids and

* Post-Doctorate Fellow (C-9717), National Cancer Institute, NIH, Bethesda, Maryland, U.S.A.
† Fellow of the Eleanor Roosevelt Foundation for Cancer Research.

vitamins and 10 per cent calf serum. For the experiments, plates were seeded at 2 to 7 $\times$ $10^5$ cells per dish.

1. *Normal Cells.*—Primary cultures were prepared from near-term foetuses (ME) of C3H or C57Bl mice or from the leg muscles (MM) of new born animals of the same strains. Secondary cultures from whole hamster embryos (HE), and cultures from a cell line (Bl 33) derived from C57Bl whole mouse foetuses were also employed.

2. *Neoplastic Cells.*—The neoplastic cell lines (all of which produced tumours after cell inoculation into animals) were derived from the following sources: (a) *Chemically induced sarcomas:* Some of these tumours were induced by a single subcutaneous injection (by syringe) of 3·0 mg. of 3,4-benzopyrene in 0·2 ml. of sesame oil or Tricaprylin, into mice of strain C3H (tumours C7 and C32), and strain C57Bl (tumour B3). Other sarcomas were induced by a single subcutaneous implantation of paraffin pellets (2–3 mm. in diameter) that contained 1 per cent 3-methylcholanthrene (tumours MC 1, and MC 2), (Prehn and Main, 1957). All of the tumours, with the exception of B3, had either not been previously transplanted *in vivo* or had been transplanted 5 or less times. The B3 tumour at the time of its use was in the 42nd *in vivo* transplant generation (b) *Polyoma virus Transformed Cells:* Bl37 and Bl T3: These cells were derived from C57Bl embryos and transformed as secondary cultures with polyoma virus (Berwald and Sachs, 1963b, unpublished data). The Bl37 cells were kept only in tissue culture, whereas the Bl T3 cells were derived from tumour tissue originating from cells transformed by polyoma *in vitro.* MT is an established cell line originally isolated from a strain SWR mouse tumour produced *in vivo* (Winocour and Sachs, 1961). T1: These cells were obtained from hamster embryo cultures transformed by polyoma *in vitro* (Medina and Sachs, 1963). (c) *Cells transformed spontaneously* in vitro: Two types of cells were obtained from cultures which had transformed spontaneously following long term passage. BHK 21: This line was originally derived from young hamster kidney tissue (Macpherson and Stoker, 1962). L Cell line: These cells were derived from mouse tissue (Earle, 1943).

*Preparation of carcinogen and non-carcinogen impregnated discs*

The carcinogens (3-methylcholanthrene and 3,4-benzopyrene) and the non-carcinogen, chrysene, were employed in the form of discs (6 mm. diameter). The discs were prepared as follows: Millipore filters (0·45 $\mu$ porosity and 25 mm. diameter) were dipped into a solution of melted paraffin (m.p. 56° C.), heated to 180°, containing 1 per cent (w/w) of the test material. Filters dipped in paraffin without any agent were used as controls. The impregnated filters were allowed to solidify and then cut into discs of uniform size. The discs were then washed in 70 per cent ethanol for 5 minutes, allowed to air dry, and stored in the dark.

*Assay of toxicity*

Cultures were grown in the presence of the test materials by floating the discs (1–2/dish) on top of the liquid medium, 10–15 minutes after seeding, or in some experiments 24 hours after cell outgrowth. The cultures were kept in an atmosphere of 5 per cent $CO_2$ in a 37° C. incubator for the duration of the experiments. During the growth of replicate cultures, the cells were observed for microscopic toxicity, and duplicate dishes were harvested for total cell counts, or for viable cell counts (with neutral red) and cell protein analysis (Alfred and Pumper, 1962).

### EXPERIMENTAL AND DISCUSSION

Experiments were designed to test whether or not cells derived from neoplastic tissues were more resistant to the toxic effects of 3,4-benzopyrene (BP) and 3-methylcholanthrene (MC), than were cell cultures obtained from normal tissues of the same host species. The results of some representative experiments are shown in Table I, in which the initial day of observed microscopic toxicity is recorded. All of the cultures derived from normal tissues and subsequently exposed to the carcinogenic hydrocarbons (2 discs/dish), showed a clear cut toxicity (rounding up, detachment, and granularity of cells) in 3–7 days. The neoplastic cells on the other hand, showed little or no toxicity to the agents tested during the culture period.

TABLE I.—*Toxicity Response of Normal and Neoplastic Cells in Culture to 3,4-benzopyrene, 3-methylcholanthrene, chrysene, and paraffin During an 8-day Period*

| Tissue origin | Tumour or cell type | Neoplastic cells. Day toxicity first appeared | | | | Normal tissue origin | Normal cells. Day toxicity first appeared | | | |
|---|---|---|---|---|---|---|---|---|---|---|
| | | BP | MC | CHR | PAR | | BP | MC | CHR | PAR |
| Chemically induced tumours | C 7 | — | — | — | — | ME | 4 | 6 | — | — |
| | MC 1 | — | — | — | — | ME | 3 | 4 | — | — |
| | MC 2 | — | — | — | — | MM | 7 | 7 | — | — |
| | C 32 | — | — | — | — | MM | 6 | 7 | — | — |
| | B 3 | — | — | — | — | MM | 5 | 4 | — | — |
| | | | | | | Bl 33 | 3 | 4 | — | — |
| Polyoma virus transformed cells | Bl 37 | — | — | — | — | HE | 3 | 3 | — | — |
| | Bl T3 | — | — | — | — | | | | | |
| | MT | — | — | — | — | | | | | |
| | T 1 | — | — | — | — | | | | | |
| Cells spontaneously transformed *in vitro* | BHK 21 | — | — | — | — | | | | | |
| | L | — | — | — | — | | | | | |

— = Little or no toxicity observed.
BP = 3,4-benzopyrene.
MC = 3-methylcholanthrene.
CHR = Chrysene.
PAR = Paraffin.

In addition to microscopic examination of the cultures, total cell counts were made at 6 to 8 days after treatment. These data (Table II), also show the differential toxicity response to the carcinogens, when comparing the normal to the neoplastic cells. The effect of BP was further studied by viable cell counts and total cell protein determination, as a function of time following carcinogen application. Replicate experiments were performed using cell cultures derived from normal embryo cells (C3H mice) and from a BP induced sarcoma (tumour C7). The results (Figs. 1 and 2) are averages from 4 sets of experiments, and the counts represent those cells which remained attached to the glass surface. Both the viable cell counts and the protein estimation showed an inhibition of growth of normal cells in response to the carcinogens and apparently no inhibition of growth in the neoplastic cell cultures. In the normal embryo cells, at 120 hours

TABLE II.—*Effect of 3,4-Benzopyrene, 3-Methylcholanthrene, Chrysene, and Paraffin on the Total Cell Number of Normal and Neoplastic Cells* in vitro *After an 8-day Period*

| | Neoplastic cells per dish | | | | | |
|---|---|---|---|---|---|---|
| | | | Count as percentage of paraffin | | | |
| Origin | Inoc. $\times 10^5$ | Count in paraffin $\times 10^5$ | BP | MC | CHR | PAR |
| C 7 | 5 | 10 | 92 | 94 | 101 | 100 |
| Bl 37 | 2 | 40 | / | 85 | 87 | 100 |
| Bl T3 | 2 | 76 | 93 | 125 | / | 100 |
| T 1 | 2 | 72 | 105 | 106 | / | 100 |
| MT | 2 | 42 | 101 | 100 | / | 100 |
| BHK 21 | 2 | 78 | 77 | 90 | / | 100 |
| L | 2 | 90 | 110 | 110 | / | 100 |
| Averages | | | 96 | 101 | 94 | 100 |

| | Normal cells per dish | | | | | |
|---|---|---|---|---|---|---|
| | | | Count as percentage of paraffin | | | |
| Origin | Inoc. $\times 10^5$ | Count in paraffin $\times 10^5$ | BP | MC | CHR | PAR |
| MM | 7 | 11 | 27 | 30 | 109 | 100 |
| MM | 7 | 7 | 60 | 68 | 127 | 100 |
| ME | 4 | 18 | 25 | 39 | 111 | 100 |
| HE | 4 | 28 | 15 | 36 | / | 100 |
| Bl 33 | 2 | 29 | / | 31 | 83 | 100 |
| Averages | | | 32 | 41 | 107 | 100 |

/ = No counts made.
BP = 3,4-Benzopyrene.
MC = 3-Methylcholanthrene.
CHR = Chrysene.
PAR = Paraffin.

of culture, the number of carcinogen treated viable cells in relation to control cells is approximately 1 : 3, whereas the total protein concentration (corrected for number of viable cells) showed a treated versus non-treated cell ratio of slightly less than 1 : 2. This indicates that at 120 hours the cell protein content in the treated cultures was higher than in the untreated control. It remains to be determined, however, whether this is related to the inhibition of cell growth in the treated cultures, or to the *in vitro* transformation manifested by a change in cell organization that has been observed after treatment with BP or MC (Berwald and Sachs, 1963a).

That the differential toxicity produced by the two carcinogens was not due to a relative resistance of neoplastic cells to toxic agents in general, was suggested by the results obtained with phenol and with formalin. The two polyoma virus-transformed (Bl 37 and Bl T3) and the two benzopyrene induced (C7 and C32) neoplastic cell lines were exposed to serial dilutions of these agents. The concentration required to produce a toxic effect was in each case identical with that required to affect non-neoplastic control cultures (HE and MM), (0·001 per cent for phenol and 0·01 per cent for formalin).

The observation that tumour cells which arose spontaneously *in vitro* or were induced by polyoma virus, chemical carcinogens, or cellophane films (Starikova and Vasiliev, 1962) were all relatively resistant to the toxic action of some carcinogenic hydrocarbons indicates that all these neoplastic cells may possess some common properties, at least in an *in vitro* environment. The results are in agreement with the supposition that carcinogenic hydrocarbons would exhibit a differential toxicity toward normal cells as compared to those derived from neoplastic tissues. Many more agents and cell types must be tested in order to ascertain the degree of generality of this phenomenon.

FIG. 1.—The effect of 3,4-benzopyrene on the growth of mouse cell cultures derived from normal embryos and BP-induced tumours.
C = non-treated control cultures.

### SUMMARY

Monolayer of normal and neoplastic cells *in vitro* were exposed to the carcinogenic hydrocarbons, 3,4-benzopyrene (BP) and 3-methylcholanthrene (MC), and to the non-carcinogenic hydrocarbon, chrysene. The normal cells were shown to be highly susceptible to the cytotoxic effects of BP and MC. On the other hand, neoplastic cells produced by these carcinogens, polyoma virus, or spontaneously transformed *in vitro* were found to be relatively resistant to the cytotoxic effects of the same carcinogens. There was no cytotoxicity of chrysene to normal or neoplastic cells.

Measurements of the total cell protein and viable cell numbers of BP-treated cultures derived from normal embryo (C3H) and BP-induced tumour (C7) tissues

Fig. 2.—Changes in cell protein content of normal embryo and BP-induced tumour cell cultures exposed to 3,4-benzopyrene.
C = non-treated control cultures.

were made. It was shown that there was an intense toxic response and a suppression of growth of the normal cells exposed to this carcinogen and little if any toxicity or inhibition of growth of the neoplastic cells.

### REFERENCES

Abell, C. W. and Heidelberger, C.—(1962) *Cancer Res.*, **22**, 931.
Alfred, L. J. and Pumper, R. W.—(1962) *Biochem. biophys. res. Commun.*, **7**, 284.
Berwald, Y. and Sachs, L.—(1963*a*) *Nature, Lond.* (In press).
Clayson, D. B.—(1962) ‘ Chemical Carcinogenesis ’, London (Churchill), p. 398.
Earle, W. R.—(1943) *J. nat. Cancer Inst.*, **4**, 165.
Haddow, A.—(1938) *J. Path. Bact.*, **47**, 581.
Macpherson, I. and Stoker, M.—(1962) *Virology*, **16**, 147.
Medina D. and Sachs, L.—(1963) *Ibid.*, **19**, 127.
Prehn, R. T.—(1963) *J. nat. Cancer Inst.* (In press).
*Idem* and Main, J. M.—(1957) *Ibid.*, **18**, 759.
Starikova, V. B. and Vasiliev, J. M.—(1962) *Nature, Lond.*, **195**, 42.
Winocour, E. and Sachs, L.—(1961) *Virology*, **13**, 207.